# Resilience enhancement interventions for disaster rescue workers: a systematic review

**DOI:** 10.1186/s13049-025-01397-0

**Published:** 2025-05-19

**Authors:** Xiaorong Mao, Ying Suo, Xiaoqing Wei, Yinxia Luo

**Affiliations:** 1 Department of Nursing, Sichuan Provincial People’s Hospital, School of Medicine, University of Electronic Science and Technology of China, Chengdu, China; 2 School of Medicine, University of Electronic Science and Technology of China, Chengdu, China; 3 Dazhou Vocational College of Chinese Medicine, Dazhou, China

**Keywords:** Disaster, Interventions, Resilience, Rescue workers, Systematic review

## Abstract

Resilience is defined as the ability of individuals to adapt to stress and adversity. In recent years, the concept of resilience in the context of disaster, particularly that of disaster rescue workers, has received considerable attention from academic researchers, disaster response organizations, and policymakers involved in disaster management. This systematic review aimed to identify interventions designed to enhance the resilience of disaster rescue workers. A systematic search was conducted from inception to January 31, 2024, in ten electronic databases: ISI Web of Science, Scopus, PubMed, MEDLINE (Ovid), Embase, Cochrane Library, CINAHL, PILOTS, PsycInfo, and the CNKI. A manual search of the reference lists of the included articles and an author search were conducted to identify additional relevant literature. A total of 22 studies that aimed to enhance resilience among disaster rescue workers were included in this review. These interventions focused on resilience-related knowledge and skills, stress and energy management, coping strategies, mindfulness, and psychological first aid. The duration of these interventions ranged from 1 to 24 h within 8 weeks, with sessions conducted in-person or online in group formats. Individual resilience, coping, social support, mindfulness, and burnout improvements were reported. The most common types of interventions were psychoeducation, followed by mindfulness-based training. However, the methodological quality of these interventions was generally sub-optimal. A well-designed intervention study is needed to enhance the resilience of disaster rescue workers.

## Introduction

The Centre for Research on the Epidemiology of Disasters (CRED) released its latest report, which found that 399 natural disasters occurred in 2023, affecting 93.1 million people [14]. Disaster relief operations inevitably expose professionals, including firefighters, healthcare workers, military personnel, and police officers—collectively referred to as rescue workers, to a range of physical hazards and trauma-related mental health challenges. This exposure puts them at high risk of negative psychological consequences [46].

It has been reported that 2.0–25.6% of rescue workers suffered from acute stress disorder (ASD) within one-month post-deployment [1]. This study suggests that the prevalence of ASD among combat soldiers may differ from that in civilian rescue workers due to variations in stress exposure and coping mechanisms while providing a reference point for understanding ASD risk in high-stress environments. According to previous studies, the prevalence rate of anxiety and depression among rescuers ranged from 9.8% to 85.2% and 11.4% to 77.8%, respectively [26]. Post-traumatic stress disorder (PTSD) has been reported in 0.4% to 46% of rescuers, with a prevalence of 8.1% to 11.9% following involvement in a disaster [10, 48]. Other health problems such as alcohol disorders, compassion fatigue, panic disorder, and sleep disturbances have also been reported [46, 60].

However, not all rescue workers suffer from the aforementioned negative psychological consequences often associated with disaster relief work. Research indicates that disaster rescuers who possess psychological resilience or have received pre-deployment psychological training are less likely to develop PTSD [3, 56], anxiety, and depression [20, 34], compassion fatigue or burnout [6] following deployment to catastrophic events. Numerous studies have demonstrated that resilience is a significant predictor of psychological distress, particularly in mitigating the adverse effects of occupational stress[11, 27].

Literature reporting on the characteristics of resilience among disaster rescue workers suggested that disaster resilience is determined by a combination of individual traits such as optimism [38], self-efficacy [38], effective stress management [15], and received social support [28, 38]. These factors collectively contribute to an individual’s ability to adapt positively and maintain psychological well-being in high-stress disaster environments. It was concluded that the resilience of disaster rescue workers reflects a combination of personal strengths and preparedness, enabling them to respond effectively to disaster events and emerge with a positive psychological state post-deployment. Studies have also suggested that resilience is not a fixed trait but rather a dynamic quality that can be enhanced through intervention programs [43], facilitating effective stress management and decision-making [33].

Studies demonstrated that resilience can be enhanced through various methods beyond direct resilience training, including coping strategies, social support, mindfulness training, and stress management. Mastering effective coping strategies provides disaster responders with broader tools and methods to address challenges in difficult situations, thereby improving individual confidence and self-efficacy [42]. Social support, particularly following exposure to traumatic events, plays a significant role in bolstering resilience. The resilience level could be fully restored to pre-stress baseline measurements in groups that received social support [40]. Mindfulness training enhances individuals’ awareness of their emotions and thoughts, helping them avoid overreacting to negative event and improving their resilience and coping ability in difficult times [29, 36]. Resilient individuals deal more effectively with adversity and the challenges of high workloads and high expectations, with high resilience associated with a lower risk of burnout[2, 52]. Stress management is a typical resilience-building training content that can directly affect how individuals cope with and recover from stress, helping individuals to better control and relieve difficulties [25, 62]. According to previous studies, social support mediates the relationship between stress and resilience.In contrast, resilience significant mediates stress and social support, and is inversely correlated with burnout. Given the interrelated and mutually reinforcing nature of coping skills, social support, mindfulness, stress management, burnout, and resilience [4, 8], assessing individual resilience can be achieved by measuring these interconnected factors.

The efficacy of existing interventions aimed at fostering psychological resilience among disaster rescue workers, however, remains inconclusive, owing to the diversity and complexity of these methodological approaches. This systematic review aims to identify and evaluate interventions designed to enhance the resilience of disaster rescuers in disaster response operations, including the details of these interventions in terms of format, approaches, training facilitators, session plans and duration, and so on. The findings of this review will provide valuable insights for researchers and organizational managers interested in developing tailored interventions to enhance the resilience of rescuers, preparing them for disaster work and protecting their mental health post-deployment.

## Methods

### Search strategy

A systematic search was conducted in ten databases, including ISI Web of Science, Scopus, PubMed, MEDLINE (Ovid), Embase, Cochrane Library, CINAHL, PILOTS, PsycInfo, and the CNKI databases, since their inception until 31 January 2024. The keywords and Medical Subject Headings (MSH) terms searched were: “emergency personnel*” or “emergency worker*” or “first responder*” or “rescue worker*” or “rescue personnel*” or “rescue*” or “disaster worker*”or “firefighter*” or “police” or “military personnel” or “emergency medical technician*” or “emergency medical services” AND “interventions” or “program*” or “therapy” or “psycho*” “strateg*”or “training” or “education” AND “resilien*” or “strength” or “hardiness” or"psychological resilienc*"or"psychological adaptation*"or"disaster resilienc*"or"positive adaptation*"AND “disaster*” or “natural disaster*” or “apocalypse” or “calamity” or “cataclysm” or “catastrophe” or “debacle” or “tragedy” or “crisis” or “crises”. Google Scholar and Dissertation Abstracts were also searched to identify further potential studies. An author search and hand search of references for the included articles were also conducted.

### Criteria for inclusion and exclusion and process of selection

Articles were selected following criteria related to population, interventions, and outcomes (PIO). Articles that: (a) focused on a population of rescue workers involved in disaster; (b) reported on interventions that aimed to enhance resilience or prevent negative psychological distress; (c) measured resilience or predictors of resilience as the primary outcome(s); and (d) were published in English or Chinese were included. Articles that: (a) focused on survivors or victims of disasters; (b) participants included both disaster rescue workers and survivors, but the data were analyzed holistically and could not be distinguished between the two groups; (c) described interventions aimed at rescuers as treatments for psychological disorders; and (d) were reported as commentaries, literature reviews, editorials, and conference proceedings were excluded.

### Data extraction and quality appraisal

The included studies in this review were extracted and tabulated with the following key information: (1) name of first author, year of publication, and country where the study was conducted; (2) study design; (3) participants and sample size; (4) intervention characteristics, such as content, session plan, duration, and format; (5) personnel who delivered the intervention; (6) instruments used and time points measured; and (7) results of the interventions.

The methodological quality of the included studies was assessed using the Mixed Methods Appraisal Tool (MMAT)–Version 2018 [14]. The MMAT has been proven to be a reliable and valid tool for assessing the methodological quality of reports of qualitative, quantitative and mixed-methods studies [14].

## Results

### Characteristics of included studies

**Fig. 1:**
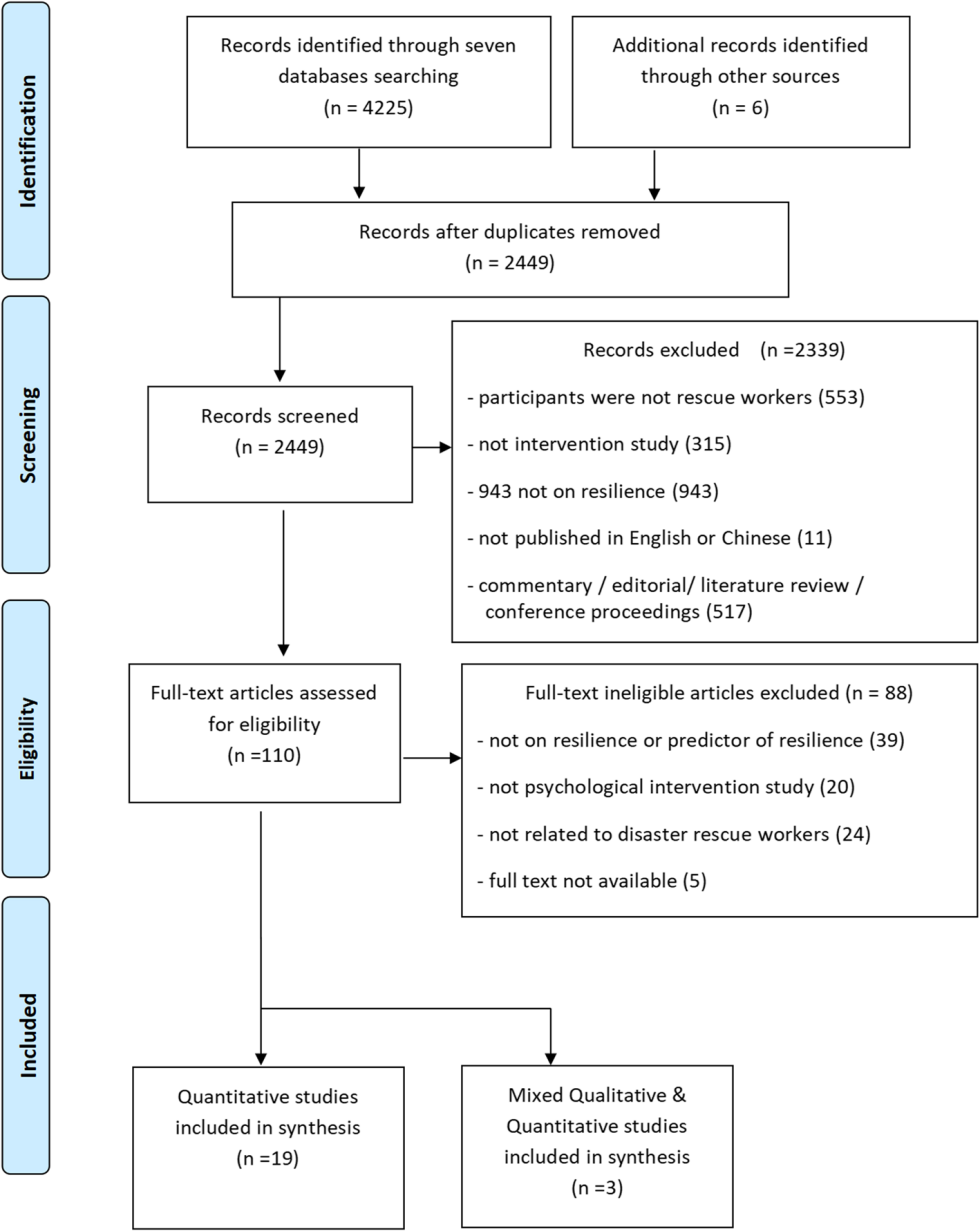
The process of literature search and selection

A total of 4231 citations were identified through the database search, and an additional six records were identified through other sources. After removing duplicates, 2449 articles were screened using titles and abstracts. A total of 4127 articles that did not meet the inclusion criteria were excluded. The remaining 110 full-text articles were assessed for eligibility. Finally, a total of 22 studies that met the inclusion criteria were included in this review. The process of selection for this review is outlined in Fig. 1***.***

A total of 22 studies, consisting of 19 quantitative studies and three mixed-method studies were included in this review. Among the 19 included quantitative studies, six were randomized controlled trials (RCTs), and 13 were non-randomized studies (NRCTs). These intervention studies were published between the year of 2008 and 2024. The included studies were primarily conducted in Western countries, with the United States of America (*n* = 12), Australia (*n* = 2), England (*n* = 1), Netherlands (*n* = 1), and Canada (*n* = 2). Other studies were conducted in Egypt (*n* = 1), mainland China (*n* = 1), Iran (*n* = 1) and Taiwan (*n* = 1). The population of rescue workers included in these studies were military personnel/soldiers (*n* = 7), healthcare workers (*n* = 6), marines (*n* = 2), police officers (*n* = 3), firefighters (*n* = 2) and mixed personnel (n = 2) involved in disastrous events. The sample size of the 22 studies ranged from 21 to 12,529, involving a total of 24,227 rescue workers. The two studies'response rates only were 48% [7] and 69%, respectively[12], and the attrition rate of these studies ranged from 0% to 59.4%. The principal reasons given for dropping out of the interventions were due to the work schedule and military redeployment [7, 12, 50] or leaving the army due to being wounded or killed in combat [37].

### Quality appraisal of included studies

The results of the quality appraisal were presented in the last column of Table 1.

**Table 1:** summary of include studies characteristics and results

Authors/ year/country	Study design	Participants	Intervention name	Training Facilitators	Measurement used and time points	Results	Quality score
Bian et al (2011) China [5]	RCT (cluster)	Military personnel: Intervention group: 201 Control group: 195	-**Coping Training Program**	Psychologist	The Coping Style Questionnaire, The Social Support Rating Scale, The Self Consistency and Congruence Scale Time 1: pre-intervention Time 2: post –intervention (immediately)	Coping strategies: problem-solving + (*p* < 0. 001), help-seeking + (*p* < 0. 001), fantasy-(*p* < 0. 001), avoidance -(*p* < 0. 001), rationalization + (*p* < 0. 05), self-blaming -(*p* ˃ 0. 05) Social support: perceived support + (*p* < 0. 001), use of support (*p* < 0. 001), objective support + (*p* ˃ 0. 05) Self-consistency: inconsistency of self and experience- (*p* < 0. 001), self-flexibility + (*p* < 0. 001), self- stereotypes -(*p* ˃ 0. 05)	**
Carr et al (2013) USA [7]	NRCT (no comparison group)	Soldiers: 189	-**Resilience Training**	Resilience trained trainer	Connor Davidson Resilience Scale, Behavior Inventory, Stress Load, Morale, And Job Performance List Time1: pre-intervention Time2: post-intervention (90 days after intervention)	Resilience: – (p = 0.033) morale: – (p = 0.007)	**
Chandra et al (2014) USA [9]	Mixed study	Medical Reserve Corps (MRC) members: 76	**-Psychological First-Aid Training**	Healthcare workers	Scale of PFA knowledge Filed notes Time1: pre-intervention Time 2: post –intervention (immediately)	perceived capability in using PFA:71% to 90% (P < 0.01) would use PFA as part of routine activities: 40% to 54% (P < 0.05) knowledge of PFA: 43% to 49% (P ˃ 0.05) participants felt more confident after the training	**
Christopher et al (2016) USA [12]	NRCT (no comparison group)	Police officers: 62	-***Mindfulness-Based Resilience Training***	Psychologist	Five Facet Mindfulness Questionnaire (FFMQ), Mindfulness Process Questionnaire (MPQ), Brief Resilience Scale (BRS), Oldenburg Burnout Inventory (OLBI), Emotional Intelligence Scale(EIS), Difficulties in Emotion Regulation Scale (DERS), Perceived Stress Scale (PSS), Global Family Functioning (GFF), Patient Reported Outcomes Measurement Information System (PROMIS) Time 1: pre-intervention Time 2: during –intervention (4 th week) Time 3:post-intervention (immediately)	Mindfulness: + (*p* < 0. 001) Resilience: + (*p* < 0. 001) Burnout:—(p < 0.001) Emotional intelligence: + (*p* = 0. 01) Emotion regulation: + (*p* = 0. 01) Stress:—(p < 0.001) Family functioning:-(*p* = 0. 12) Fatigue: -(p < 0.001)	**
Cohn et al (2008) Australia [13]	RCT	Soldiers Intervention group: 101 Control group: 73	-***Cognitive-Behavioral Program***	Psychologist	Real Events Attributional Style Questionnaire, Brief Cope, positive state of mind(PSOM), General Health Questionnaire-12(GHQ-12) Time1: pre-intervention Time 2: post-intervention (immediately) Time 3: follow up (3 weeks after intervention)	Attribution stability and globality + (p < 0.05) Coping: self-blame: -(p < 0.001) Psychological adjustment: + (*p* < 0. 001)	**
Hammermeister et al. [23] USA	NRCT (no comparison group)	Soldiers: 27	-***Mental Skills Training***	Army officer	The Ottawa Mental Skills Assessment Tool, *Self-Esteem Rating Scale* Time 1: pre-intervention Time 2: post –intervention (immediately)	Self—confidence: + (*p* = 0.0004) Self esteem: increased from T1(M = 78.85, SD = 31.74) to T2(M = 89.82, SD = 26.73 (p = 0.003)	**
Johnson et al. [29] USA	NRCT	Marines: Intervention group:147 Control group:134	-** Mindfulness-Based Mind Fitness Training**	Mindfulness trained trainer	Response to Stressful Experiences Scale Time 1: pre-intervention Time 2: post –intervention (immediately) Time 3: after stress exposure (immediately)	After stress exposure Heart rate recovery greater (p < 0.001) Breathing rate recovery greater (p < 0.001) As resilience increased, insula activity decreased (r = −0.42, p < 0.05)	**
Kaplan et al (2017) USA [30]	NRCT (no comparison group)	Law enforcement officers (LEOs):47 firefighters (FFs):22	-**Mindfulness-Based Resilience Training**	Psychologist	The Brief Resilience Scale, The Five Facet Mindfulness Questionnaire-Short Form, The Oldenburg Burnout Inventory Time 1: pre-intervention Time 2:Post-intervention (immediately)	Resilience: + (*p* < 0.001) Mindfulness: + (*p* < 0.001) Burnout:—(*p* < 0.001)	*
Ke et al (2017) [32] Tai Wan	NRCT (no comparison group)	Healthcare providers:67	**-Psychological Support**	Psychologist	Immediate Self-Administered Questionnaire Time 1: pre-intervention Time 2:Post-intervention (1 month)	Incidence of posttraumatic psychiatric disorders: 16.4% to 0% (p < 0. 05)	*
Lester et al (2011) USA [37]	NRCT	Soldiers: Intervention group: 12,529 Control group: 9,479	**-Master Resilience Training**	Resilience trained trainer	Global Assessment Tool: resilience and psychological health (R/PH) Time 1: pre-intervention Time 2:post-intervention (immediately) Time 3: follow up (6 months after intervention)	Good coping: + (p = 0.01) Friendship: + (p = 0.00) positive affect: + (p = 0.293) family fitness: + (p = 0.293372) spiritual fitness: + (p = 0.852) negative affect:—(p = 0.445) Catastrophic thinking:—(p < 0.01) Depression:—(p = 0.10) Age was a moderator of MRT training in a number of different aspects of Soldier fitness: more effective for Soldiers between the ages of 18–24	**
Marks et al., [47] USA	NRCT (no comparison group)	Firefighter and emergency workers:30	**- Recognize, Evaluate, Advocate, Coordinate, and Track training**	Psychologist	Knowledge questionnaire, REACT PSP Self-Effcacy Questionnaire, Generalized Self-Effcacy Scale, The Brief Resilience Scale Time 1: pre-intervention Time 2:Post-intervention (immediately) Time 3: follow up (2 weeks after intervention) Time 4: follow up (3 months after intervention)	Score of knowledge: + (p = 0.03, η2 =.209) REACT PSP self-efficacy: + (p <.001, η2 =.577) general self-efficacy: (pre: M = 31.75, SD = 3.60; post: M = 32.78, SD = 2.76; 2 weeks: M = 34.00, SD = 3.35; and 3 months: M = 33.14, SD = 3.24) resilience: (pre: M = 22.38, SD = 4.47; post: M = 23.50, SD = 4.26; 2 weeks: M = 24.33, SD = 3.50; and 3 months: M = 24.00, SD = 3.87)	**
Maunder et al. [49] Canada	NRCT (no comparison group)	Healthcare workers:158(short course 51, medium course 54, long course 53	- **Computer-Assisted Resilience Training**	Healthcare worker	Questionnaire of Confidence in Supporting and Training, Pandemic Self-Efficacy Scale, Inventory of Interpersonal Problems (IIP-32), Ways of Coping Inventory Time 1: pre-intervention Time 2:Post-intervention (immediately)	Confidence in Supporting and Training: + (*p* < 0.001) Pandemic Self-efficacy: + *p* < 0.001) Coping: + (*p* ˃0.05) the medium length course was the optimal duration	**
Meulen et al. [50] Netherland	NRCT	Police officers: Intervention group:74 Control group:96	***- Mental Strength Training***	Psychologist	Mental Toughness Questionnaire-48 (MTQ-48), Resilience Scale (RS), Symptoms Check List 90-R (SCL-90-R), Self-Rating Inventory for PTSD (SRIP) Time 1: pre-intervention Time 2:Post-intervention (3 months) Time 3: follow up (9 months after intervention)	Resilience MTQ-48: + (p = 0.002, Cohen’s d = 0.26) RS: + (p = 0.044, Cohen’s d = 0.01) Anxiety, depression, and PTSD: no significant difference	**
Powell et al. (2016) USA [55]	Mixed study	Healthcare workers:69	***-Psycho-educational intervention***	Social worker	Professional Quality of Life Scale, Perceived Stress Scale, the Stress Arousal Checklist, the Social Provisions Scale, the Ways of Coping tool, the Coping Self Efficacy Scale Time 1: pre-intervention Time 2:Post-intervention (immediately) Time 3: follow up (3 weeks after intervention)	Professional Quality of Life: + (*p* ˃ 0.05) Perceived Stress: + (*p* ˃ 0.05) Social Provisions: + (*p* < 0.05) Coping Self Efficacy: + (*p* ˃ 0.05) Perceived knowledge: + (*p* < 0.001) Social support and team building was enhanced	**
Skeffington et al (2016) Australia [57]	RCT (cluster)	Firefighters Intervention group:30 Control group:45	- ***Mental Agility and Psychological Strength Training Program***	Psychologist	Knowledge of trauma checklist, the traumatic stress schedule, The PTSD Checklist—Civilian Version, the depression, anxiety, and stress scale, the Brief Coping Orientations to Problems Experienced (Brief COPE) scale Time 1: pre-intervention Time 2:Post-intervention (6 months after intervention) Time 3: follow up (12 months after intervention)	Trauma knowledge: + (*p* <.001, η2 =.14) Stress:—(*p* = 0.113, η2 = 0.02) PTSD: + (*p* = 0.001, η2 =.08) Anxiety:- (*p* = 0.507,η2 = 0.007) Depression:—(p = 0.017, η2 = 0.04) Perceived social support:—(*p* = 0.004, η2 = 0.06) Social support satisfaction:—(*p* =.223, η2 = 0.02) Adaptive coping:—(*p* = 0.804, η2 = 0.00) Maladaptive coping:—(*p* = 0.811, η2 = 0.00)	**
Stanley et al. (2011) USA [59]	Mixed study	Marines: Intervention group:34 Control group:21	-**Mindfulness-Based Mind Fitness Training**	Army officer	Five Facet Mindfulness Questionnaire, Perceived Stress Scale, Personal Outlook Scale Time 1: pre-intervention Time 2:Post-intervention (immediately)	Mindfulness: + (between high practice group and low practice group, *p* < 0.01; between high practice group and comparison group, *p* = 0.05) Stress:—*(p* > 0.16) Mindfulness negatively associated with perceived stress (r = −0.46, *p* < 0.01)	**
Ebrahimian et al. [17] Iran	NRCT	Emergency medical services personnel: Intervention group:n = 32 Control group:n = 32	-***Hotwash***	Psychologist	an EMSRS questionnaire Time 1:pre-intervention Time 2:a day after the psychological hotwash Time 3:six weeks after the psychological hotwash	One day after the intervention: Job motivation: + (*p* = 0.040) Communication challenges:-(*p* = 0.442) Social support: + (*p* = 0.029) Remaining calm:-(*p* = 0.526) Self-management:-(*p* = 0.102) Consequences of stress:-(*p* = 0.422) Six weeks after the intervention: Job motivation:-(*p* = 0.193) Communication challenges:-(*p* = 0.261) Social support: + (*p* = 0.003) Remaining calm:-(*p* = 0.152) Self-management:-(*p* = 0.059) Consequences of stress:-(*p* = 0.547)	**
Mahaffey et al (2021) USA [42]	RCT	Disaster workers: Intervention group:n = 78 Control group:n = 89	***-The Disaster Worker Resilience Training (DWRT) Program***	Psychologist or social worker	Health‑promoting lifestyle profile II (HPLP II) Perceived stress scale (PSS) PTSD checklist for DSM-5 (PCL-5) Patient health questionnaire (PHQ-9) Life events checklist for DSM‑5 (LEC-5) Time 1:pre-intervention Time 2:post-intervention (3 months after intervention)	Healthy lifestyle behaviors: + (η2 = 0.03; *p* = 0.03) Stress management: + (η2 = 0.03, *p* = 0.04) Spiritual growth: + (η2 = 0.03, *p* = 0.02)	**
Wild et al (2020) England [61]	RCT	Emergency workers: Resilience intervention:n = 317 Psychoeducation:n = 113	-***Resilience intervention and Psychoeducation***	Trained staff	***Primary outcome:*** -The Warwick Edinburgh Mental Wellbeing scale -The Connor-Davidson Resilience Scale -The General Self-Efficacy Scale -The Social Participation scale and the Social Support scale adapted from Sarason et al.’s ***Secondary outcome:*** The Depressive Attributions Questionnaire The Brief Coping Behaviour Questionnaire An unpublished trauma screener The PCL-5 The PHQ-9 The General Anxiety Disorder 7 (GAD-7) The Alcohol Use Disorders Identification Test Time 1:pre-intervention Time 2:post-intervention (immediately) Time 3:at three-month follow-up	Resilience (CD-RISC):Baseline Mean(SD) = 66.49 (14.72),Post Mean(SD) = 67.94 (17.01),Follow-up Mean(SD) = 68.52(16.18) Social Support (Home):Baseline Mean(SD) = 33.04 (6.08),,Post Mean(SD) = 33.64 (6.43),,Follow-up Mean(SD) = 34.14 (6.71) Social Support (Work):Baseline Mean(SD) = 27.20 (6.64),,Post Mean(SD) = 27.17 (6.58),Follow-up Mean(SD) = 27.42 (6.80)	**
Eweida et al (2023) Egypt [19]	NRCT	pre-licensure nursing students: Intervention group:n = 31 Control group:n = 33	***-Psychological first aid***	Not mention	-The General Health Questionnaire (GHQ-12) -Abridged Connor-Davidson Resilience Capacity Scale-10 (CD-RISC-10) Time 1:pre-intervention Time 2:post-intervention (immediately) Time 3:at two-months follow-up	reduction in the psychological distress levels: + (*p* = 0. 001) silience capacity level: + (*p* = 0.019)	**
Heyen et al (2021) Switzerland [24]	NRCT	first responders: n = 52	***-COAST***	Not mention	-the Perceived Stress Scale -the 9-item Patient Health Questionnaire -the 7-item Generalized Anxiety Disorder Scale -the General Self-Efficacy Scale -the Posttraumatic Stress Disorder Checklist of the Diagnostic and Statistical Manual of Mental Disorders Time 1:at baseline Time 2:at 2 weeks’ follow-up Time 3:at 4 weeks’ follow-up	number of clicks on each module: mean activity score = 15; SD = 11.11	**
Fikretoglu et al (2019) Canada [21]	RCT	Military recruits Intervention group:n = 1452 Control group:n = 1379	***-The Road to Mental Readiness (R2MR)***	Psychologist	-Abridged Connor-Davidson Resilience Capacity Scale-10 (CD-RISC10) Time 1: at baseline Time 2: at 5 weeks Time 3: at 9 weeks	Resilience (CD-RISC) total score: −0.14 (*p* = 0.41) GAD total score: − 0.10 (*p* = 0.66)	**
**Note**:/Met 0% of MMAT criteria; * Met 25% of MMAT criteria; ** Met 50% of MMAT criteria. RCT: randomized controlled trials, NRCT: non –randomized controlled trials; + : positive effect; -: negative effect							

According to the Mixed Methods Appraisal Tool (MMAT) criteria [14], while 20 out of 22 included studies obtained a quality score of 50% [5, 7, 9, 12, 13, 17, 19, 21, 23, 24, 29, 37, 42, 47, 49, 50, 55, 57, 59, 61], the other two were 25% [30, 32]. The quality of the 20 studies was fair, and two were relatively poor.

Twenty-two studies were included in this review, all meeting the inclusion criteria. However, several methodological limitations need to be highlighted. The randomized controlled trials [5, 13, 17, 19, 42, 57] did not report the randomization process or allocation concealment, which may introduce selection bias and affect the validity of the results. The non-randomized controlled trials exhibited various limitations, such as not recruiting participants in a manner that minimizes selection bias [37], failing to describe the appropriateness of measurements [12, 30, 32], lacking a control group [7, 9, 12, 23, 24, 30, 32, 47, 49], or not achieving an acceptable response rate of 60% or above [7, 37]. The mixed methods studies [9, 55, 59] did not explain the rationale for integrating qualitative and quantitative methods to answer the research question, which may limit the interpretability and coherence of the findings.

### Characteristics of interventions

A summary of the characteristics of these interventions are outlined in Table 2.

**Table 2:** Summary of intervention characteristics of the included studies

Authors/ year/country	Approach	Content	Session Plan	Duration	Format
Bian et al (2011) China [5]	Training of coping strategy	***-Survival skills:*** basic knowledge and skills of survive,potential danger of emergency tasks,personalities of emergency tasks, ***-Self-regulation skills:*** cognitive and appraisal methods ***-Communication and relationship skills:*** skills of help-seeking to others ***-Stress management:*** self-management of stress emotion ***-Coping strategies***	−2 h each session per week	−14 weeks	- lecture, individual practice, self-introspection, homework, diaries, discussion
Carr et al (2013) USA [7]	Resilience training	***-Self-regulation skills:*** optimism, self-efficacy, self-regulation, empathy,emotional awareness ***-Coping strategies:***problem-solving, flexibility ***-Communication and relationship skills:*** relationships	-not presented	−12 weeks	-did not present format
Chandra et al (2014) USA [9]	Psychological education/support	***-Psychological first aid:***listen to the individual and understand both verbal and nonverbal cues; protect the individual by determining realistic ways to help and provide reassurance, support, and encouragement;connect the individual to family, friends, and resources in the community *(LPC)* ***-Knowledge of resilience:***knowledge of resilience, disaster, stress	−2 h for LPC, time of workshop did not report	−1 day	-slide presentation, video scenarios, workshop, role -play, discussion
Christopher et al (2016) USA [12]	Mindfulness-based intervention	***-Mindful awareness and movement:***mental focus, sustained attention and a broad sense of personal and situational awareness	−2 h per week (the seventh week:6 h)	−8 weeks	-individual exercise, didactic, discussion, debriefing and homework
Cohn et al (2008) Australia [13]	Congnitive-behavioral program	***-Self-regulation skills:*** cognitive restructuring ***-Coping strategies***	−40 min each session	−6 weeks(2 sessions)	-lecture
Hammermeister et al. [23] USA	Psychological education/support	***-Self-regulation skills:*** mental skills foundations, self-confidence, goal-setting, attention control, life-coaching theory, team building -***Energy management***	−1.5 h per module	−12 h (8 modules)	-did not present format
Johnson et al. [29] USA	Mindfulness-based intervention	***-Self-regulation skills:*** attention control, tolerance of present-moment experiences, ***-Stress management:***self-regulation of the stress response	−2 h per week and 4 h workshop	−8 weeks	-individual exercise, didactic, and homework
Kaplan et al (2017) USA [30]	Mindfulness-based intervention	***-Mindful awareness and movement:***body scan, sitting and walking meditations, mindful movement, and other MBSR practices	−2 h per week (the seventh week:6 h)	−8 weeks	-experiential, didactic, discussion and assigned homework
Ke et al (2017) [32] Tai Wan	Psychological education/support	***-Psychological first aid:*** knowledge and skills of Psychological First-Aid ***-Relaxation skills*** ***-Self-regulation skills:*** expressions of feelings	-not presented	-not presented	-mini-lectures and debriefing
Lester et al (2011) USA [37]	Resilience training	***-Self-regulation skills:*** self-awareness, self-regulation, optimism, mental agility, create positive emotion ***-Knowledge of resilience:*** concept of resilience ***-Stress management&energy management*** ***-Communication and relationship skills:*** build strengths character and stronger relationship	−8 h per day	−10 days	-didactic, discussion
Marks et al (2017) [47] USA	Psychological education/support	***-Stress management:*** knowledge of posttraumatic stress disorder (PTSD) and acute stress disorder (ASD), stress injury, skills of stress management ***-Communication and relationship skills:*** communication skills	-not presented	−6-h session totally	-didactic, workbook, question-and-answer, role-play
Maunder et al. [49] Canada	Resilience training	***-Coping strategies:*** coping training, disaster preparation ***-Self-regulation skills:*** balancing family and word, managing drugs and alcohol, danger signals and resources for getting help ***-Knowledge of resilience*** ***-Stress management*** ***-Relaxation skills***	-participants completed the course in several sittings at their own pace	-short course:1.75 h; medium course:3 h; long course:4.5 h	-audio and video mini-lectures, printed fact sheets, interactive reflective exercises
Meulen et al. [50] Netherland	Psychological education/support	***-Self-regulation skills:*** skills of goal setting, attention control,self-confidence ***-Stress management:*** stress reactions ***-Energy management***	−8 h per day	−3 days	-lecture, exercises
Powell et al. (2016) USA [55]	Psychological education/support	***-Stress management:*** knowledge of stress, common reactions to a traumatic event, job burnout and compassion fatigue ***-Coping strategies:*** coping strategies and support	-not presented	−3 h totally	-not presented
Skeffington et al (2016) Australia [57]	Psychological education/support	***-Stress management:*** information about stress, PTSD ***-Self-regulation skills:*** social support, self care, meaningful connection ***-Coping strategies***	−1 h per week	−4 weeks	- didactic, group discussion
Stanley et al. [59] USA	Mindfulness-based intervention	***-Stress management:*** stress resilience skills, information about stress and traumain the body ***-Knowledge of resilience***	−2 h per week and a full day	−8 weeks	-didactic, group discussion, exercises, outside class practice using CDs
Ebrahimian et al (2021) [17] Iran	Psychological education/support	***-Communication and relationship skills:*** create a platform for all group members to speak and participate by asking them to introduce themselves	−2 h for the first session,between 70 and 90 min later	−1 month	-lecture, group discussion
Mahaffey et al (2021) USA [42]	Resilience training	***-Stress management:*** recognize signs and symptoms of disaster work-related stress ***-Communication and relationship skills:*** obtain support through employer and community resources ***-Coping strategies:*** build resilience by using coping strategies	-not presented	−4-h session totally	-digital presentation,workshop
Wild et al (2020) England [61]	Resilience training	***-Stress management:*** stress management and mindfulness tools for reducing stress ***-Mindful awareness and movement:*** psycho-education includes six topics: sleep, stress, depression, anger, mindfulness, and post-traumatic stress disorder	−2.5 h per session per week for resilience intervention,one topic released each week for psychoeducation	−6 weeks	-online module, group-based course, homework exercises
Eweida et al (2023) Egypt [19]	Psychological education/support	***-Self-regulation skills:*** enriching with information, practicing cognitive reframing, installing future orientation, delaying any life-altering decisions or changes ***-Communication and relationship skills:*** enlisting family and friends’ support ***-Stress management***	-two times per week, with each session taking one hour	−10 sessions totally	-group discussion, didactic
Heyen et al (2021) Switzerland [24]	Mindfulness-bad intervention	***-Self-regulation skills:*** online modules targeting self-efficacy, sleep quality, mindfulness, gratitude and positive reframing ***-Mindful awareness and movement:*** online modules targeting mindfulness	- not presented	−15 weeks	-online module
Fikretoglu et al (2019) Canada [21]	Congnitive-behavioral program	***-Self-regulation skills:*** increase mental health literacy, change attitudes and intentions towards mental health service use (MHSU) ***-Stress management:*** teach stress management skills	-not presented	−13 weeks	-exercises

The interventions adopted approaches such as psychological education/support (*n* = 9) [9, 17, 19, 23, 32, 47, 50, 55, 57], mindfulness-based intervention (*n* = 5) [12, 24, 29, 30, 59], resilience training (*n* = 5) [7, 37, 42, 49, 61], cognitive-behavioral program (*n* = 2) [13, 21], and training of coping strategy (*n* = 1) [5]. Most interventions were delivered face-to-face in a group by psychologists (*n* = 11), resilience/mindfulness trained trainers (*n* = 4), army officers (*n* = 2), healthcare workers (*n* = 2), and social workers (*n* = 1). At the same time, the two studies did not mention the person who delivered the intervention [19, 24].

The interventions were delivered at various time points related to disaster deployment, some before deployment (*n* = 6), the majority during deployment (*n* = 12), and fewer after deployment (*n* = 4). There was considerable variation in the dosage and duration of these interventions. For the psychological education/support interventions, one study provided three consecutive days of 8-h psychological education sessions; another study offered an initial 2-h session followed by sessions ranging from 70 to 90 min for one month; two studies comprised sessions of 1 to 1.5 h each over a period of 4 to 8 weeks; four studies provided psychoeducation ranging from 2 to 10 h in total; one study did not report the dosage and duration of the intervention. The four mindfulness-based training studies were offered for two hours per session for 8 weeks, with an extended course of 4 to 8 h in the sixth or eighth week, while another online study lasted for 15 weeks with no dosage specified. For resilience training, two studies did not specify dosages but lasted for four sessions and 12 weeks, respectively; one study provided 8-h lectures for 10 days; another consisted of 2.5 h per session per week for 6 weeks; one offered courses lasting from 1.75 to 4.5 h with a flexible completion schedule. The one study that used a cognitive-behaviour approach consisted of two 40-min sessions for 6 weeks, while the dosage was not mentioned in another study. The one study of a coping training programme redundant consisted of 2 h each session per week for 14 weeks. The dosage and duration of these interventions are summarized in Table 2.

The effectiveness of interventions was measured immediately (*n* = 15), 1 month (*n* = 3), 3 months (*n* = 3), and 6 months (*n* = 1) after completion of interventions. Only ten of the 22 included studies reported follow-up assessments, with evaluation periods ranging from two weeks to 12 months after the intervention.

The most common intervention was didactic instruction (*n* = 16). Others were group discussion (*n* = 6), skills practice (*n* = 6), work assigned to do at home (*n* = 5), doing worksheets (*n* = 2), role-play (*n* = 2), and debriefing approach (*n* = 2). Three studies did not describe the format of their interventions [7, 23, 55].

### Content of resilience enhancement intervention

The content of the interventions mainly included stress management [5, 19, 21, 23, 29, 30, 37, 42, 49, 55, 57, 59, 61], coping strategies [5, 7, 13, 42, 49, 55, 57], information about resilience [9, 19, 37, 49, 59], mindfulness awareness [12, 24, 29, 30, 59, 61], and self-regulation skills [7, 17, 19, 24, 29, 37, 57]. Other psychological skills or knowledge such as communication and relationships [7, 9, 37], energy management [23, 37, 50], relaxation skills [32, 49], psychological first aid [9, 32], and survival skills [5], were adopted in the studies.

### Effectiveness of the interventions

The effectiveness of these interventions was measured using a range of outcome measures, such as resilience, coping, social support, mindfulness, stress, and burnout. Resilience was designated as the primary outcome measure, while others were categorized as secondary outcomes. The characteristics and psychometric properties (including reliability and validity) of the relevant scales employed in each study are summarized in Table 3.

**Table 3:** Summary of measuring instruments of the included studies

**Resilience**								
**Name of Measuring Instrument**	**Domains**	**Number of Items**		**Rating Scale**	**Validity in Instrument**	**Reliability in Instrument**	**Study**	**Internal Reliability Measures Across Included studies**
the Connor Davidson Resilience Scale (CDRS)	ability to tolerate painful experiences	−25 items/10 items		−5-point Likert scale	*-Content validity*: not reported *-Construct validity*: Analysis of data from subjects in the general population sample yielded five factors whose eigenvalues were, respectively, 7.47, 1.56, 1.38, 1.13, and 1.07. -*Convergent Validity*: negative correlation with the Perceived Stress Scale (PSS-10) (Pearson *r* = −0.76, *P*<.001); negative correlation with the Sheehan Disability Scale (SDS) (Pearson r= −0.62, P <.0001); the Sheehan Social Support Scale (SSS) correlated significantly with the CD-RISC(Spearman r =0.36, P<.0001). -*Discriminant Validity*: not significantly correlated with the ASEX at baseline (*r*= −0.34, *P*=.11) or at endpoint (*r*= −0.30, *P*=.21)	***-Internal Consistency****:* Cronbach’s α for the full scale was 0.89 for Group 1 and item-total correlations ranged from 0.30 to 0.70. *-Test-retest*: The mean (sd) CD-RISC scores at time 1 [52.7 (17.9)] and time 2 [52.8 (19.9)] demonstrated a high level of agreement, with an intraclass correlation coef ficient of 0.87.	Carr et al. (2013) [7]	α=0.89
							Eweida et al. (2023) [19]	α=0.88
							Fikretoglu et al. (2019) [21]	T1: α=0.85 T2: α=0.91 T3: α=0.93
							Wild et al. (2020) [61]	α=0.93
the Brief Resilience Scale (BRS)	perceived stress, depression, and active coping	−6 items		−5-point Likert scale	*-Content validity:* not reported *-Construct validity: *The results for each sample revealed a one-factor solution accounting for 55–67% of the variance (Samples 1–4 = 61%, 61%, 57%, 67%, respectively). The loadings ranged from.68 to.91. -*Convergent Validity*: positively correlated with the resilience measures, optimism, social support, active coping, positive reframing and purpose in life, and negatively correlated with pessimism, alexithymia, negative interactions, behavioral disengagement, denial, and self-blame. -*Discriminant Validity*: “resilience” measures were almost always related in the expected direction with the outcomes, with the exception that ego resiliency was only marginally related to less negative affect.	*-Internal Consistency:* Internal consistency was good, with Cronbach’s alpha ranging from 0.80–0.91 *-Test-retest*: The BRS was given twice in two samples with a test-retest reliability (ICC) of.69 for one month in 48 participants from Sample 2 and.62 for three months in 61 participants from Sample 3.	Christopher et al. (2016) [12]	Pre-MBRT：α=0.87 Post-MBRT：α=0.90
							Kaplan et al. (2017) [30]	Pre-MBRT：α=0.87 Post-MBRT：α=0.90
							Marks et al. (2017) [47]	Not report
the Emergency Medical Services Resilience Scale (EMSRS)	job motivation, communication challenges, social support, calmness at the incident scene, self-management or self-care, and consequences of stress	−31 items		−5-point Likert scale	*-Content validity:* 13 items were omitted (P < 0.63) and the SCVI/Ave was 0.96. In the CVI assessment, four items were omitted. The factor analysis with varimax rotation was used and 10 items were eliminated due to their lack of compatibility with the desired factor. *-Construct validity: *Six factors had values higher than one. This six-factor structure accounted for 51.82% of the total variance.	*-Internal Consistency*: The internal consistency of the scale was calculated with a Cronbach's alpha coefficient of 0.91 and a theta coefficient of 0.97. The ICC of the EMSRS was 0.851 and the ICC of its dimensions ranged from 0.72 to 0.87. *-Test-retest*: ICC=0.851	Ebrahimian et al. (2021) [17]	α=0.91
Resilience Scale(RS)	personal competence and acceptance of self and life	−25 items		−5-point Likert scale	*-Content validity*: not reported *-Construct validity*: The various items loaded onto six different factors with an Eigenvalue greater than 1, which confirms that the RS can have a five or even a six-factor structure. -*Convergent &Discriminant Validity: *There was a significant moderate positive correlation between the entire RSnl and the BRSnl, and a significant moderate positive correlation between the entire RSnl and the AAQ II, and there was a significant strong positive correlation between the BRSnl and the AAQ II.	*-Internal Consistency*: Cronbach’s alpha ranges from.87 to.95.	Meulen et al. (2017) [50]	α=0.93
the Mental Toughness Questionnaire-48 (MTQ-48)	Challenge, Commitment, Control, and Confidence	−48 items		−5-point Likert scale	*-Content validity*: not reported *-Construct validity*: S-Bχ^2^ (1074) = 2599.046, p <.001, RCFI =.623, RNNFI =.604, SRMR =.070, RMSEA =.054, 90% CI [.052,.057]	*-Internal Consistency*: The scale demonstrated adequate internal reliability for the subscales of commitment, confidence abilities and confidence interpersonal, but not for challenge, control-emotion and control-life.	Meulen et al. (2017) [50]	α=0.91
the General Health Questionnaire-12 (GHQ-12)	psychological distress and social dysfunction factors	−12 items		−4-point Likert scale	*-Content validity*: not reported *-Construct validity*: The validity of the GHQ as shown by its linear associations with independent clinical assessments (typically r = 0.70 or greater). The factor structure of the GHQ have typically yielded a large general factor, with three more subsidiary ones.	*-Internal Consistency & Test-retest*: The development studies showed that the full scale exhibited high internal consistency and good retest reliability over a period of 6 months.	Cohn et al. (2008) [13]	α=0.87–0.89
the Response to Stressful Experiences Scale (RSES)	meaning-making and restoration, active coping, cognitive flexibility, spirituality, and self-efficacy	−22 items		−5-point Likert scale	*-Content validity*: All corrected item-total correlations, again for 20 of the 22 items, exceeded 0.45. *-Construct validity*: These 5 factors accounted for over 53% of the total variability in item responses. -*Convergent Validity*: Correlating moderately to moderately high (coeffi cients of 0.61 and 0.81) with scores on the CD-RISC, and with DRS-15 was only 0.38. -*Discriminant Validity* Weakly related to individual differences in responding to highly stressful events, including scores on the Combat Experiences Scale (coeffi cients of 0.01, −0.18, and 0.02) and MMPI-2 RF Lie Scale (coefficient of 0.19). *-Concurrent Validity*: Those scoring higher on the RSES tended to score higher on measures of Unit Support (coeffi cient of 0.38) and Postdeployment Social Support (coeffi cients of 0.36 and 0.56).	*-Internal Consistency & Test-retest*: The resulting 22-item scale demonstrated sound internal consistency (a = 0.91–0.93) and good tes--retest reliability (r = 0.87).	Johnson et al. (2014) [29]	Not reported
the Global Assessment Tool (GAT)	emotional, family, social, spiritual fitness and organizational context	−140 items		−5-point Likert scale	*-Content validity*: not reported *-Construct validity*: Items cohered as intended into the domains of concern to the CSF program. Spiritual fitness items (e.g., “My life has a lasting meaning”) and family fitness items (e.g.,“My family supports my decision to serve in the Army”) respectively loaded on and indeed defined their own separate factors. -*Convergent Validity*: In almost all cases, items derived from a given scale converged with one another.	*-Internal Consistency*: Alpha coefficients for scales exceeding.80	Lester et al. (2011) [37]	α>0.80
the Immediate Self-Administered Questionnaire	recurrent and intrusive distressing recollections of the event, including images, thoughts, or perceptions; tachycardia; muscle tension; difficulty relaxing; difficulty falling or staying asleep; feeling fear; feeling guilty; needing help after the medical response; and needing to talk with someone in private	−9 items		-yes or no option for each item	*-Content*: The items from 1 to 7 are based on the Diagnostic and Statistical Manual of Mental Disorders, Fouth Edition, Text Revision.The eighth and ninth items are the active needs from healthcare providers.	Not reported	Ke et al. (2017) [32]	Not reported
**Coping**								
**Name of Measuring Instrument**	**Domains**	**Number of Items**		**Rating Scale**	**Validity in Instrument**	**Reliability in Instrument**	**Study**	**Internal Reliability Measures Across Included studies**
the Brief COPE	active coping, planning, positive reframing, acceptance, humour, religion, emotional support, instrumental support, self-distraction, denial, venting, substance use, behavioural disengage- ment, and self-blame	−28 items		−4-point Likert scale	*-Content validity*: not reported *-Construct validity*: all items contribute with their respective factor with loadings greater than the recommended minimum of 0.40. -*Convergent & Discriminant Validity:* Higher coefficients are observed between instrumental support and emotional support (r = 0.65) and between active coping and planning (0.56).	*-Internal Consistency*: Cronbach’s alpha for the total scale is adequate exceeds the minimum value of 0.60.	Cohn et al. (2008) [13]	α>0.50
							Skeffington et al. (2016) [57]	α=0.74–0.96
the Coping Style Questionnaire	problem-solving, self-blaming, helpseeking, fantasy, avoidance and rationalization.	−62 items		−2-point Likert scale	*-Content validity*: not reported *-Construct validity*: The six factors with eigenvalues greater than were extracted, and then the absolute value of the factor load above 0.35 (including 0.35) was proposed to form six homogeneous coping factors.	*-Internal Consistency*: not reported *-Test-retest: *The retest correlation coefficients are: R1=0.72; R2=0.62; R3=0.69;R4=0.72;R5=0.67;R6=0.72.	Bian et al. (2011) [5]	α=0.75–0.89
the Ways of Coping Inventory	problem-solving and seeking support	−66 items		−4-point Likert scale	*-Content validity*: not reported *-Construct validity*: The 27 items classified as problem-focused, 21, or 78%, correlated more strongly with the first empirical factor. Of the 41 items classified as emotion-focused, 28, or 68%, were correlated more strongly with the second empirical factor. -*Convergent & Discriminant Validity: *The correlations between the P- and E-scales in these administrations were.35 (N = 81),.52 (N = 63), and.44 (N = 83). The mean correlation was.44.	*-Internal Consistency*: The mean alpha coefficient for the two adminis- trations of the P-scale was 0.80 and for the E- scale,0.81.	Maunder et al. (2010) [49]	α=0.73
**Social Support**								
**Name of Measuring Instrument**	**Domains**	**Number of Items**		**Rating Scale**	**Validity in Instrument**	**Reliability in Instrument**	**Study**	**Internal Reliability Measures Across Included studies**
the Social Support Rating Scale	objective support, perceived support, and the use of support	−10 items		−4-point Likert scale	*-Content validity*: not reported *-Construct validity*: not reported -*Predictive Validity: *There was a moderate correlation between the scale prediction outcome and the physical health outcome.	*-Internal Consistency*: not reported *-Test-retest: *The total score consistency was R=0.92 (P<0.01), and the consistency of each item was between 0.89 and 0.94.	Bian et al. (2011) [5]	α=0.89–0.93
the Social Provisions Scale	Seeking Social Support, Planful Problem Solving, and Positive Reappraisal	−66 items		−5-point Likert scale	*-Content validity*: not reported *-Construct validity*: Factor analysis has confirmed a six-factor structure that corresponds to the six social provisions. The six social provisions in combination accounted for 66% of the variance in scores on the UCLA Loneliness Scale. -*Discriminant Validity: *Analyses of data from a college student sample have supported the discriminant validity of the Social Provisions Scale against relevant measures of mood (e.g., depression), personality (i.e., neuroticism, introversion-extraversion), and social desirability.	*-Internal Consistency*: Internal consistency for the total scale score is relatively high, ranging from.85 to.92 across a variety of populations. Alpha coefficients for the individual subscales range from.64 to.76.	Powell et al. (2016) [55]	α=0.92
the Social Participation scale and the Social Support Scale	social support in home and work	−10 items totally		−7-point/3-point Likert scale	The Social Participation scale: *-Content validity:* not reported *-Construct validity: *The individual items of the DFI have been shown to reliably load on a single factor. **The Social Support Scale: **Detailed information is provided in the table below.	The Social Participation scale: *-Internal Consistency*: The Cronbach's a for the present sample was 0.80. **The Social Support Scale: **Detailed information is provided in the table below.	Wild et al. (2020) [61]	Social Participation：α=0.92 Social Support (Home)：α=0.77 Social Support (Work)：α=0.83
the Emergency Medical Services Resilience Scale (EMSRS)	*-Social Support:* social support	−31 items		−5-point Likert scale	*-Content validity*: 13 items were omitted (P < 0.63) and the SCVI/Ave was 0.96. In the CVI assessment, four items were omitted. The factor analysis with varimax rotation was used and 10 items were eliminated due to their lack of compatibility with the desired factor. *-Construct validity*: Six factors had values higher than one. This six-factor structure accounted for 51.82% of the total variance.	*-Internal Consistency*: The internal consistency of the scale was calculated with a Cronbach's alpha coefficient of 0.91 and a theta coefficient of 0.97. The ICC of the EMSRS was 0.851 and the ICC of its dimensions ranged from 0.72 to 0.87. *-Test-retest*: ICC=0.851	Ebrahimian et al. (2021) [17]	α=0.91
the Social Support Questionnaire	*-Social Support: *availability and satisfaction	−6 items		−6-point Likert scale	*-Content validity*: not reported *-Construct validity*: Two factors were found to account for 71, 14% of the overall variance. All the items load onto their original subscales with loading values of 0.70 or greater in each case.	*-Internal Consistency*: Cronbach’s alpha coeffi cients of reliability of the total SSQ6 is 0.885.	Skeffington et al. (2016) [57]	Not reported
**Mindfulness**								
**Name of Measuring Instrument**	**Domains**	**Number of Items**	**Rating Scale**		**Validity in Instrument**	**Reliability in Instrument**	**Study**	**Internal Reliability Measures Across Included studies**
the Five Facet Mindfulness Questionnaire	*-Mindfulness:*observing, describing, acting with awareness, nonjudging of inner experience, and nonreactivity to inner experience	−39 items	−5-point Likert scale		*-Content validity*: not reported *-Construct validity*: Results of the initial EFA yielded 26 factors with eigenvalues greater than 1.0 and accounting for 63% of the total variance. Facet loadings for the final model differed, on average, by only two one hundredths (.02). Fit indices for this model were CFI =.96, NNFI =.94, and RMSEA =.07 -*Convergent &Discriminant Validity: *All correlations were in the expected directions, and all but one (MQ with openness to experience) were statistically significant.	*-Internal Consistency*: The following alpha coefficients were obtained for the five mindfulness questionnaires, suggesting good internal consistency: MAAS =.86, FMI =.84, KIMS =.87, CAMS =.81, MQ =.85 (ns = 595–613).	Christopher et al. (2016) [12]	Pre-MBRT：α =0.82 Post-MBRT：α =0.88
							Kaplan et al. (2017) [30]	Pre-MBRT：α =0.82 Post-MBRT：α =0.88
							Stanley et al. (2011) [59]	Not reported
**Stress and Burnout**								
**Name of Measuring Instrument**	**Domains**	**Number of Items**		**Rating Scale**	**Validity in Instrument**	**Reliability in Instrument**	**Study**	**Internal Reliability Measures Across Included studies**
the Perceived Stress Scale (PSS)	*-Stress and Burnout:*how often participants experience specific thoughts, feelings, or difficulties related to stress	−4 items		−5-point Likert scale	*-Content validity*: not reported *-Construct validity*: the two-factor structure for the PSS-14 accounted for less than 50% of the total variance. -*Criterion Validity: *PSS was strongly correlated with only the mental component of health status as measured by the Medical Outcomes StudyeShort Form 36.	*-Internal Consistency*: The reported Cronbach’s alpha was <.70 in half of the six studies in which the PSS-4 was evaluated. *-Test-retest reliability* :The test-retest reliability of the PSS was assessed in met the criterion of >.70.	Christopher et al. (2016) [12]	Pre-MBRT：α =0.69 Post-MBRT：α=0.68
							Mahaffey et al. (2021) [42]	α=0.88–0.91
							Powell et al. (2016) [55]	α=0.85
							Stanley et al. (2011) [59]	Not reported
Generalized Anxiety Disorder Scale (GAD-7)	*-Stress and Burnout:*anxiety level	−7 items		−4-point Likert scale	*-Content validity*: not reported *-Construct validity: *There was a strong association between increasing GAD-7 severity scores and worsening function on all 6 SF-20 scales. -*Convergent &Discriminant Validity: *The Beck Anxiety Inventory (*r* = 0.72) and the anxiety subscale of the Symptom Checklist-90 (*r* = 0.74). The GAD-7 correlated most strongly with mental health (0.75), followed by social functioning (0.46), general health perceptions (0.44), bodily pain (0.36), role functioning (0.33), and physical functioning (0.30).	*-Internal Consistency*: Cronbach α =.92. *-Test-retest reliability *: intraclass correlation = 0.83	Fikretoglu et al. (2019) [21]	T1: α=0.87 T2: α=0.89 T3: α=0.92
the Emergency Medical Services Resilience Scale (EMSRS)	*-Stress and Burnout:*calmness at the incident scene and consequences of stress	−31 items		−5-point Likert scale	*-Content validity*: 13 items were omitted (P < 0.63) and the SCVI/Ave was 0.96. In the CVI assessment, four items were omitted. The factor analysis with varimax rotation was used and 10 items were eliminated due to their lack of compatibility with the desired factor. *-Construct validity*: Six factors had values higher than one. This six-factor structure accounted for 51.82% of the total variance.	*-Internal Consistency*: The internal consistency of the scale was calculated with a Cronbach's alpha coefficient of 0.91 and a theta coefficient of 0.97. The ICC of the EMSRS was 0.851 and the ICC of its dimensions ranged from 0.72 to 0.87. *-Test-retest*: ICC=0.851	Ebrahimian et al. (2021) [17]	α=0.91
the Depression Anxiety Stress Scales (DASS)	*-Stress and Burnout:*depression, anxiety and stress	−42 items/21 items		−4-point Likert scale	*-Content validity*: not reported *-Construct validity*: GFI= 0.92、AGFI=0.90，NFI=0.87、CFI =0.88、IFI =0.88、RFI =0.85、TLI =0.86，RMSEA = 0.065. -*Convergent &Discriminant Validity: *There was a moderate correlation among the dimensions (0.577–0.691), and a high correlation between the dimensions and the total scale (0.805–0.897).	*-Internal Consistency*: The internal consistency of the depression, anxiety, and stress subscales was 0.77, 0.79, and 0.76, respectively, and the internal consistency coefficient of the total scale was 0.89.	Skeffington et al. (2016) [57]	Not reported
the Old Lenbuth Burnout Inventory (OLBI)	*-Stress and Burnout:*exhaustion and disengagement	−16 items		−4-point Likert scale	*-Content validity*: not reported *-Construct validity*: The fitting index of the model did not reach the 0.90 standard, and subscales exhaustion and disengagement had an estimated correlation of.52. -*Convergent Validity: *All items of Exhaustion (of both instruments) loaded significantly on an Exhaustion factor, while the items of Cynicism and of Disengagement had significant loadings on an Attitudes factor.	*-Intercorrelation*: All scales exhibited reliabilities greater than 0.70.	Christopher et al. (2016) [12]	Pre-MBRT：α = 0.85 Post-MBRT；α = 0.88
							Kaplan et al. (2017) [30]	Pre-MBRT：α = 0.85 Post-MBRT；α = 0.88

### Primary outcome

#### Resilience

Of the 22 studies in this review, more than half (*n* = 13) directly assessed the effects of interventions on resilience. Various instruments were used to measure resilience: the Connor Davidson Resilience Scale (CDRS)[7, 19, 21, 61], the Brief Resilience Scale (BRS) [12, 30, 47], the Emergency Medical Services Resilience Scale (EMSRS)[17], the Resilience Scale (RS), the Mental Toughness Questionnaire-48 (MTQ-48) [50], the General Health Questionnaire-12 (GHQ-12) [13], the Response to Stressful Experiences Scale (RSES) [29], the Global Assessment Tool (GAT) [37], and the Immediate Self-Administered Questionnaire [32].

An RCT study conducted in Australia showed that the soldiers who received a brief cognitive-behavioral training program (CBT) reported better psychological adjustment from baseline measure (T1) to immediately (T2) and 3 weeks (T3) after the interventions.The group receiving CBT reported an increase in positive affect of mind from T1 to T3, with M ± SD = 12.90 ± 2.74, 12.58 ± 3.03, 13.93 ± 3.43 respectively, *P* <0.001, λ^2^ = 0.87; and a decrease in distress from T1 to T3, with M ± SD = 10.95 ± 5.44, 11.50 ± 6.26, 8.79 ± 6.55 respectively, *P* <0.05, λ^2^ = 0.95), compared to the control group [13]. This finding aligns with a quasi-experimental study conducted in the Netherlands, which demonstrated an increase in resilience scores following mental health training [50]. Another study of healthcare rescue workers showed a decrease in the incidence of post-traumatic psychiatric disorders, considered a predictor of psychological resilience, after receiving psychological support (decreased from 16.4% to 0%, *P* < 0. 05) [32].

Four studies in the USA reported significant benefits from interventions on resilience enhancement.Three studies using mindfulness-based training significantly increased the resilience scores among those who participated in the programmes (*P<*0.05) [12, 29, 30]. Another study involving 22,278 soldiers demonstrated that those who received 10 days of Master Resilience training had greater resilience as measured 6 months after training (emotional fitness: 1.31% increase, ŋ2 = 0.02; social fitness: 0.66% increase, ŋ2 = 0.02, optimism: 1.02% increase, ŋ2 = 0.01) [37].

However, one study of 189 soldiers showed that the scores of resilience, as measured by the Connor-Davidson Resilience Capacity Scale(CD-RISC), decreased from a mean of 77.6 ± 13.0 to 74.2 ± 16.6 before and 90 days after training (*P* = 0.033) [7]. Another study in England showed no significant difference between the group who received the resilience intervention group and the control group on any outcome measure, either post-intervention or at follow-up time points. The resilience intervention group reported increasing scores on the CD-RISC immediately after the intervention and at 3 months (pre: M ± SD = 66.49 ± 14.72; post immediately: M ± SD = 67.94 ± 17.01; and 3 months: M ± SD = 68.52 ± 16.18), the psychoeducation group showed a similar trend (pre: M ± SD = 67.48 ± 14.62; post immediately: M ± SD = 68.48 ± 15.26; and 3 months: M ± SD = 69.43 ± 15.25)[61]. Another psychological hotwash study reported that there was a statistically significant difference in mean resilience scores between the intervention and control groups one day after the program (*P* = 0.003).However, no difference was found after 6 weeks, which was probably due to the session interruption [17].

Another study of 30 first responders (firefighters and emergency workers) suggested that there were some improvements in resilience score, as measured by the Brief Resilience Scale (BRS), after receiving exercises of stress management and communication training (pre: M ± SD = 22.38 ± 4.47; post immediately: M ± SD = 23.50 ± 4.26; 2 weeks: M ± SD = 24.33 ± 3.50; and 3 months: M ± SD = 24.00 ± 3.87), but the results were not analyzed by the authors from a statistic perspective due to the limited statistical power observed [47].

In short, 13 out of 22 included studies found mixed results regarding the impact of interventions on resilience among uniformed rescuers, including police officers, healthcare workers, firefighters, and soldiers. However, the evidence on which interventions are effective in increasing the resilience of disaster responders remains inconsistent. Therefore, more scientific and targeted interventions need to be developed to increase responders'resilience and help them cope better with different types of disasters.

### Secondary outcome

#### Coping

The effects of interventions on coping were explored in five studies. The measurements used to assess coping were: the Brief Cope [18, 57], the Coping Style Questionnaire [5], and the Ways of Coping Inventory [49].

A study of soldiers reported higher adaptive coping scores of 1.3% after receiving resilience training (*P* = 0.001), while no change in maladaptive coping was found [37]. Another study of Chinese military personnel who attended a 28-h coping strategies training programme reported that their adaptive coping strategies such as support seeking (d_2_-d_1_ = −0.122, *P* < 0.001), and problem-solving, were improved (d_2_-d_1_ = −0.077, *P* < 0.001). Meanwhile, no significant difference was found for coping through self-blame (*P* > 0.05) [5]. In another study, among the soldiers who received an 80-min cognitive-behavioral program, more of them had a decreased score of self-blame three weeks after the intervention, compared with the control groups (29% vs. 10%, *P* < 0.01) [13].

Interventions targeting coping strategies do not always yield significant improvements. A study involving firefighters who received a 4-h psychological strength training programme found no significant differences in both adaptive (* P* = 0 0.804, η^2^ = 0.00) and maladaptive coping (* P* = 0 0.811, η^2^ = 0.00) between the intervention and control groups on the Time × Condition interaction [57]. Similarly, another study of healthcare workers who received computer-assisted resilience training did not exhibit significant changes in coping strategies. Specifically, support-seeking coping showed no significant difference (pre vs. Post- intervention: M ± SD = 1.5 ± 0.7 vs. 1.4 ± 0.6, *P* = 0.95), problem-solving coping remained unchanged (pre vs. post intervention: M ± SD = 1.5 ± 0.7 vs. 1.5 ± 0.7, *P* = 0.40), and escape-avoidance (denial) coping (pre vs. post intervention: M ± SD = 0.6 ± 0.5 vs. 0.6 ± 0.5, *P* = 0.06), also did not show significant changes [49].

Therefore, no conclusions can be drawn about the effectiveness of interventions to improve coping strategies.

### Social support

Only five of the 22 included studies assessed the effectiveness of interventions on social support. The instruments used were the Social Support Rating Scale [5], the Social Provisions Scale [55], the Social Participation scale and the Social Support Scale [61], the Emergency Medical Services Resilience Scale (EMSRS) [17], and the Social Support Questionnaire [57].

In a study of military personnel who underwent coping training that included a social support module, significant increases were observed in perceived social support scores (pre vs. post intervention: M ± SD = 19.46 ± 3.89 vs. 21.28 ± 3.42, *P* < 0. 001), and the use of support (pre vs. post intervention: M ± SD = 8.55 ± 2.06 vs. 9.42 ± 1.67, *P* < 0. 001) [5]. However, no significant differences were found in objective support (pre vs. post intervention: M ± SD = 9.19 ± 2.62 vs. 9.43 ± 2.58, *P* > 0. 05). Another study in Iran reported statistically significant differences in social support resilience scores between the hotwash group and the control group from one day after intervention (T1) to six-week follow-up (T2)(T1: *P* = 0.029; T2: *P* = 0.003) with the hotwash group performed better [17]. However, baseline differences between the groups reduced confidence in the results. A psychoeducation intervention study conducted in the USA among healthcare workers showed an increase in social provision from pre-intervention to 3-week follow-up (M = 27.34 vs. 28.39, *P* < 0.05) [55]. Among Australian firefighters who received psychological training, a significant increase was found in perceived social support (*P* = 0.004, η2 = 0.06). However, no significant differences were observed in social support satisfaction (* P* = 0.223, η2 = 0.02) [57].

However, a study of emergency workers in England who received a resilience intervention (pre vs. post intervention: M ± SD = 33.04 ± 6.08 vs. 33.64 ± 6.43) found no significant difference in social support (* P* = 0.246) compared with those receiving psychoeducation (pre vs. post intervention: M ± SD = 32.58 ± 6.86 vs. 32.83 ± 7.09) [61]. Therefore, it is concluded that perceived social support can be improved in military personnel, firefighters, and healthcare workers who received psycho-interventions.

### Mindfulness

Four of the 22 studies examined the effects of interventions on mindfulness, and one of these studies focused on participant acceptance, activity, and feedback [24]. Mindfulness was assessed in all three studies using the Five Facet Mindfulness Questionnaire [12, 30, 59].

All three studies showed a significant positive effect of the interventions on mindfulness. One study reported a marked increase in mindfulness scores (not reacting to inner experience, not judging inner experience, and acting with awareness) among those who completed the 8-week training, with significant differences between before and after the interventions (M ± SD: T1 = 46.18 ± 6.89, T3 = 55.16 ± 7.89), *P* < 0.01). In contrast, no significant difference was found during the intervention’s fourth week (M ± SD: T2 = 48.56 ± 6.49, *P* ˃ 0.10) [12]. Another study found that mindfulness scores significantly improved post-intervention, particularly among those who dedicated more time to practicing mindfulness skills (pre-intervention vs. post-intervention: 131.60 vs. 139.60, *P* < 0.05) [59]. It has also been found that increased mindfulness was related to increased resilience (*b* = 0.41, SE = 0.11, *P* < 0.01), which implicated the indirect effect of resilience on mindfulness [30]. In contrast, a study utilizing an online psychoeducation platform measured participant engagement through module click-through rates (mean activity score = 15; SD = 11.11), but did not employ scales to assess mindfulness levels pre- and post-intervention, focusing instead on feasibility and adherence [24].

It is concluded that mindfulness intervention programmes can improve mindfulness in rescue workers, which in turn enhances their resilience to some extent.

### Stress and burnout

Four studies measured stress using the Perceived Stress Scale (PSS) [12, 42, 55, 59], the Generalized Anxiety Disorder Scale (GAD-7) [21], the Emergency Medical Services Resilience Scale (EMSRS) [17], and the Depression Anxiety Stress Scales (DASS) [57]. Two studies examined burnout, both of which used the Old Lenbuth Burnout Inventory (OLBI) [12, 30].

A study provided police officers with 2-h weekly mindfulness-based resilience training for 8 weeks, resulting in a statistically significant decrease in Perceived Stress Scale (PSS) scores from the start of training (T1) to the fourth week (T2) and immediately after training (T3) (M ± SD: T1 = 9.70 ± 2.73, T2 = 9.29 ± 2.59, T3 = 7.67 ± 2.57, *P* < 0.001, effect size = 0.75) [12]. Another study involving healthcare workers who attended a 3-h psychoeducation workshop reported a significant reduction in acute stress scores from pre-intervention to immediately post-intervention (M ± SD = 10.53 ± 7.14 vs. 6.78 ± 5.42 respectively, *P* < 0.001) [55]. Nevertheless, the score of perceived stress increased at the 3-week follow-up, with M ± SD = 14.93 ± 6.46 vs.15.49 ± 6.93 respectively, *P* ˃ 0.05.

However, a study of marines who received 2-h per week for 8 weeks of mindfulness-based mind-fitness training reported no significant differences in perceived stress between the intervention and control groups (*P* ˃ 0.16) [59]. While there is another study indicating that firefighters who were offered psychological strength training reduced their stress scores between pre-intervention and 12-month follow-up (M ± SD = 6.50 ± 0.87 vs. 6.11 ± 1.03, *P* = 0 0.858, η2 = 0.006) [57].

Two studies examined the effectiveness of interventions on levels of burnout among rescue workers. One study of 43 police officers who completed an 8-week mindfulness-based resilience training program reported a decrease in OLBI scores from the beginning (T1:pre-intervention) to the mid-point (T2:at 4 th week) and end of the training (T3:immediately post-intervention) (M ± SD: T1 = 39.19 ± 6.87, T2 = 37.88 ± 2.596.71, T3 = 33.89 ± 7.44, *P* < 0.001, effect size = 0.74) [12]. In another study of law enforcement officers and firefighters who received 8 weeks of mindfulness-based resilience training, the scores of OLBI also decreased significantly (M ± SD: pre vs. post training: LEOs 39.18 ± 6.98 vs. 34.36 ± 7.38, FF 37.43 ± 6.85 vs. 31.90 ± 7.50, *P* < 0.001) [30]. It is worth noting that neither of these studies included comparison groups.

In summary, the available evidence suggests that it is uncertain whether interventions reduce stress and alleviate occupational burnout among disaster responders. More research is needed in the future to further test the effectiveness of these interventions.

## Discussion

The purpose of this systematic review was to identify existing interventions for resilience enhancement and to examine in detail the approaches, delivery person, content dosage and duration, format, delivery time points, the evaluation time points, and the effectiveness of these interventions.

A total of 22 studies were included in this review. More than half of the included studies (12/22, 54.5%) were conducted during deployment. The overarching objectives of these interventions were to enhance resilience and to prevent psychological health problems, with the exception of treating psychiatric diseases.

The findings of this review suggest that these interventions may be beneficial in enhancing the resilience of disaster rescue workers. Our study aligns with previous studies indicating that resilience may play a mediating role in the effects of mindfulness on psychological symptoms such as depression and anxiety [53]. Furthermore, empirical evidence demonstrates that resilience functions as a critical mediator in multiple psychosocial domains, including the established positive association between social support level and resilience development and its buffering effects on mental health challenges such as loneliness and burnout [39]. These findings are consistent with those from previous reviews of resilience training programs, which also demonstrated improvements in resilience among various adult demographics, including university students, cancer patients, and caregivers [16, 31, 41].

The most frequently adopted approach in these 22 included intervention studies was psychological education/support, followed by mindfulness-based intervention and resilience training. However, the conclusion of this study is not definitive as to which approach is more effective. Using multiple instruments to assess the same outcomes reduces the possibility of conducting a meta-analysis to compare the effectiveness of these interventions. Therefore,the necessity for future research is highlighted, as well as conducting multi-arm studies to validate and compare the effectiveness of different interventions, and thereby determining the most efficacious form of intervention.

The review of the extant literature on interventions to reduce perceived stress reveals an absence of consensus regarding the efficacy of such approaches. However, a review and meta-analysis suggest that work-related stress can be mitigated through mindfulness-based stress reduction and cognitive-behavioral training [51]. This may be explained by the time participants spent training [58]. Consequently, it is imperative that future studies focusing on stress reduction among rescue workers should consider the appropriate session plan and duration.

None of the studies incorporated within the current review adopted a conceptual framework to guide the development of their respective resilience enhancement interventions. It is important for intervention evaluation research, as the conceptual framework is instrumental in guiding the development of the intervention and elucidating its mechanism [35]. Developing a model or framework of resilience in the context of disaster among rescue workers will not only provide researchers with a comprehensive understanding of the relationships between various concepts and the mechanism of resilience, but will also guide researchers in developing tailored interventions accordingly.

The methodological quality of the included studies was limited. The Mixed Methods Appraisal Tool (MMAT) is a critical appraisal tool designed to evaluate the methodological quality of studies with diverse designs, including qualitative, quantitative, and mixed-methods research. The quality scores of all the included studies were found to be less than 50%, with two studies receiving scores as low as 25% according to the MMAT criteria. One of the studies [30] that was considered as poor quality was the only study among the 22 that adopted the Brief Resilience Scale to measure the concept of resilience directly as an outcome measure. Another study [32] with low methodological quality only met 25% of MMAT criteria, with two assessment items remaining inadequately answered. Specifically, this study failed to provide sufficient information to address the following criteria: (a) the appropriateness of measurements concerning both outcomes and interventions and (b) the completeness of the outcome the data. In line with the overall aim of conducting a systematic and comprehensive evidence synthesis, and in accordance with the MMAT methodological framework [14], which explicitly discourages the exclusion of studies based on methodological limitations alone, we ultimately decided to retain these two studies for analysis, despite their acknowledged methodological limitations. Moreover, of the 22 included studies, only six were randomized control trials, and comparison groups were not designed in 10 studies. As such, there is inadequate evidence to definitively conclude that these interventions have effectively enhanced the resilience of disaster rescue workers. More scientific intervention studies with randomized controlled trials design should be developed and implemented among rescue workers to enhance their resilience, which will benefit their psychological well-being after a disaster.

### Recommendation for organizations involved in disasters

Managers of organizations involved in disaster response play a critical role in supporting rescue workers, both physically and psychologically. Pre-deployment training is essential to build resilience among rescue workers, helping them adapt to adverse events and challenging work environments while mitigating the risk of negative psychological outcomes. Research has highlighted the importance of providing psychological and material resources to individuals in high-stress roles [45], and studies show that rescue workers who receive greater support from their communities or units report higher job satisfaction and lower levels of psychiatric symptoms [63]. Therefore, it is suggested that supervisors should establish a good relationship with rescue workers and try their all best to understand what the rescue workers really want and provide reasonable and appropriate services such as psychological support to the rescue workers as long as they need it. 

### Recommendations for resilience training program for disaster workers

This systematic review highlights the critical need for well-designed resilience intervention studies targeting disaster rescue workers, particularly in China, which ranks among the top five countries most frequently affected by natural disasters [22]. Disaster rescue workers, especially nurses, were found to be more susceptible to negative psychological sequelae than other professional rescue workers [46]. Resilience training may be beneficial for workers recruited as rescue personnel to prevent them from experiencing negative mental health outcomes following disaster rescue work.

Conducting resilience interventions prior to deployment is crucial for disaster responders. Research indicates that individuals who undergo comprehensive disaster preparedness training are more likely to develop an internal locus of control, which fosters confidence in managing emergencies at disaster sites [44, 54].

The content of the intervention should be multi-dimensional, including knowledge of resilience, stress management, coping strategies, mindfulness skills, psychological first aid (PFA), and relaxation skills. The whole intervention can be arranged around 1.5 to 2 h each week and lasting 6 weeks in a face-to-face group format. The outcome used to evaluate the effectiveness of the intervention should be comprised of resilience, coping, and psychological health outcomes, including general stress, anxiety, and depression, which can be assessed before training, immediately after training, and at least 3-month follow-up.

### Limitations

This systematic review has several limitations. Firstly, despite efforts to locate as many eligible studies as possible, no Chinese-language articles were found that met the inclusion criteria, and the studies included were only published in English. Several articles in other languages were excluded, inevitably leading to some language bias. Secondly, unpublished articles or gray literature were not included, giving rise to publication bias in this systematic review, which may not provide a whole picture of this topic. Thirdly, a wide range of scales were used to assess outcomes, including comprehensive psychological scales, which may reduce the accuracy and reliability of the measurements. Finally, articles aimed at preventing psychological health problems and improving psychological well-being were included, of which the outcomes that do not directly measure resilience were included. In order to ensure the comprehensiveness of the search, studies considered to be of poor quality were also included. This may affect the comparability of outcome assessment and views on the effectiveness of these interventions by downgrading the quality of the evidence and increasing the heterogeneity in outcome analysis.

### Implications

The results of this review provide several key implications for future nursing practice and research. Firstly, long-term, longitudinal interventions that examine more diverse populations and intervention effects are necessary. The paucity of high-quality randomized controlled trials necessitates the conducting additional multi-arm studies in the future to validate and compare the effectiveness of different interventions to determine the best form of intervention. Secondly, while various exercise programs have been implemented to explore differences in intervention duration and frequency, large-scale meta-analyses are urgently needed to identify the effects on different populations and outcomes and to establish the most effective interventions. Thirdly, the components of the programs should be studied, the intervention content that can effectively improve the resilience level of rescuers should be determined, and individualized intervention programs should be developed for different groups.

Therefore, the findings of this review highlight that tailored, context-specific interventions can provide important support for increasing the resilience of disaster responders. However,it is critical to continually assess, improve, and develop strategies for these interventions to meet changing needs in healthcare settings.

## Conclusion

This review summarizes 22 studies on interventions to enhance the resilience of disaster responders. Psychological support and mindfulness interventions have significantly enhanced resilience and improved social support for rescuers. Combining online video viewing and face-to-face intervention is an effective intervention method. Follow-up and ongoing support are essential to the effectiveness of the intervention. The overall improvement can be achieved by developing an intervention plan that is tailored to the characteristics of the rescuer's work. However, the interpretative validity of these findings needs to be assessed with caution, given the pervasive methodological limitations of the included studies. This review makes important points and highlights the need for more high-quality, large-sample study designs in the future to draw conclusive results.

## Data Availability

No datasets were generated or analysed during the current study.
